# Transcriptional Profiling of Swine Lung Tissue after Experimental Infection with *Actinobacillus pleuropneumoniae*

**DOI:** 10.3390/ijms140510626

**Published:** 2013-05-21

**Authors:** Zhicai Zuo, Hengmin Cui, Mingzhou Li, Xi Peng, Ling Zhu, Ming Zhang, Jideng Ma, Zhiwen Xu, Meng Gan, Junliang Deng, Xuewei Li, Jing Fang

**Affiliations:** 1 College of Veterinary Medicine, Sichuan Agricultural University, Ya’an 625014, Sichuan, China; E-Mails: zzcjl@126.com (Z.Z.); pengxi197313@163.com (X.P.); abtczl72@126.com (L.Z.); abtcxzw@126.com (Z.X.); gm0320@163.com (M.G.); dengjl213@126.com (J.D.); fangjing4109@163.com (J.F.); 2 Laboratory of Animal Disease and Human Health, Sichuan Agricultural University, Ya’an 625014, Sichuan, China; 3 College of Animal Science and Technology, Sichuan Agricultural University, Ya’an 625014, Sichuan, China; E-Mails: mingzhou.li@163.com (M.L.); zhm3000@163.com (M.Z.); jideng_ma@sina.com (J.M.); xuewei.li@sicau.edu.cn (X.L.)

**Keywords:** porcine pleuropneumonia, infection, injury, *Actinobacillus pleuropneumoniae*, agilent porcine genechip, microarray analyses, cytokine, host defense response

## Abstract

Porcine pleuropneumonia is a highly contagious respiratory disease that causes great economic losses worldwide. In this study, we aimed to explore the underlying relationship between infection and injury by investigation of the whole porcine genome expression profiles of swine lung tissues post-inoculated with experimentally *Actinobacillus pleuropneumoniae*. Expression profiling experiments of the control group and the treatment group were conducted using a commercially available Agilent Porcine Genechip including 43,603 probe sets. Microarray analysis was conducted on profiles of lung from challenged *versus* non-challenged swine. We found 11,929 transcripts, identified as differentially expressed at the *p* ≤0.01 level. There were 1188 genes annotated as swine genes in the GenBank Data Base. GO term analysis identified a total of 89 biological process categories, 82 cellular components and 182 molecular functions that were significantly affected, and at least 27 biological process categories that were related to the host immune response. Gene set enrichment analysis identified 13 pathways that were significantly associated with host response. Many proinflammatory-inflammatory cytokines were activated and involved in the regulation of the host defense response at the site of inflammation; while the cytokines involved in regulation of the host immune response were suppressed. All changes of genes and pathways of induced or repressed expression not only led to a decrease in antigenic peptides presented to T lymphocytes by APCs via the MHC and alleviated immune response injury induced by infection, but also stimulated stem cells to produce granulocytes (neutrophils, eosinophils, and basophils) and monocyte, and promote neutrophils and macrophages to phagocytose bacterial and foreign antigen at the site of inflammation. The defense function of swine infection with *Actinobacillus pleuropneumoniae* was improved, while its immune function was decreased.

## 1. Introduction

Porcine pleuropneumonia (PP) is a highly contagious respiratory disease that causes great economic losses worldwide [1]. The disease, which occurs in swine of all ages, is highly infectious, often fatal, and characterized by necrotizing, hemorrhagic bronchopneumonia and serofibrinous pleuritis [1]. *Actinobacillus pleuropneumoniae* (APP) is the causative agent of PP and can spread quickly by air-borne particles and/or touching a contaminated surface, and often kills infected animals in the acute phase when extensive lung hemorrhage and necrosis occur. Swine that survive often develop pleurisy, the sequelaes of local necrosis of the pleura, or became healthy carriers of APP.

The porcing lung infected with APP has previously been reported to result in local production of proinflammatory proteins or to mRNA encoding the cytokines interleukin (IL)-1α, IL-1α, IL-6 and the chemokine IL-8 [2]. Likewise, bioactive protein and/or mRNA code IL10, IL12p35, TNF-α and INF-α have shown to be up-regulated after infection with APP *in vivo* or *in vitro* [2–4]. Using cDNA microarrays, Moser and co-workers found 307 anonymous transcripts in blood leukocytes from swine that were significantly affected by experimental infection with APP [5]. Hedegaard *et al.* investigated the molecular characterization of the early response in pigs to experimental infection with APP serotype 5B, using cDNA microarrays [6]. In this study, two-colour microarray analysis was conducted to identify genes being significantly differently expressed in non-inflamed lung tissue compared with inflamed lung tissue sampled from the same animal [6]. The samples of lung tissue were studied by manual hybridization to the pig array DIAS_PIG_27K2 that contains 5375 PCR products amplified from unique cDNA clones [6]. Hedegaard and co-workers found three subsets of genes consistently expressed at different levels depending upon the infection status, and a total of 357 genes differed significantly in their expression levels between infected and non-infected lung tissue from infected *versus* non-infected animals [6]. Mortensen *et al.* studied the local transcriptional response in different locations of lung from pigs experimentally infected with the respiratory pathogen APP 5B, using porcine cDNA microarrays (DJF Pig 55 K v1) representing approximately 20,000 porcine genes printed in duplicate [7]. Within the lung, Mortensen and co-workers found a clear division of induced genes as, in unaffected areas a large part of differently expressed genes were involved in systemic reaction to infections, while differently expressed genes in necrotic areas were mainly concerned with homeostasis regulation [7]. However, a limited number of genes relative to the whole Porcine Genome have been studied in previous documents by using cDNA microarrays [5–7]. Thus, transcriptional profiling of whole porcine genome in lung tissue sampled from inoculated *versus* non-inoculated swine would lead to greater knowledge of the host response dynamics to bacterial infection in the lung. This knowledge is important to obtain a more complete picture of the lung-specific host reactions in the pathogenesis of respiratory infection.

In the present study, the Agilent Whole Porcine Genome Oligo (4 × 44 K) Microarrays (one-color platform), which is a commercially available Agilent Porcine Genechip that included 43,603 probe sets, were used to detect the changes in gene expression of infected pigs’ lungs from non-inoculated animals. Ten transcripts (top six up-regulated and top four down-regulated in microarray data) were selected to verify the accuracy and reproducibility of the microarray data by real-time qRT-PCR.

## 2. Results

### 2.1. Clinical Symptoms and Necropsy Findings

The symptoms of lung lesions in the TG were typical after swine infected with APP. Swine showed hyperthermia (40.6–42.0 °C), dyspnea and anorexia after inoculation with APP 24–48 h. Two swine died with respiratory distress at post-inoculation 36–48 h. In the autopsy, the lungs were found to be severely damaged by acute, multifocal, fibrino-necrotizing and hemorrhagic pneumonia complicated by acute diffuse fibrinous pleuritis. The tracheobronchial lymphoid nodes were enlarged and congested.

**Figure 1:**
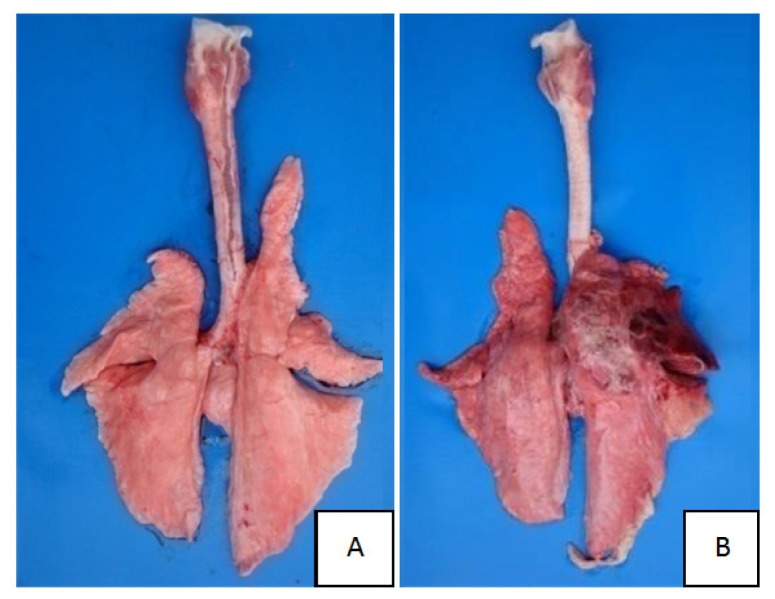
(**A**) Normal lung from healthy swine; (**B**) Damaged lung after APP infection (Lung in TG showing swelling, bleeding and fibrinous exudate sticking to the lung surface; while no lesion in CG).

**Figure 2:**
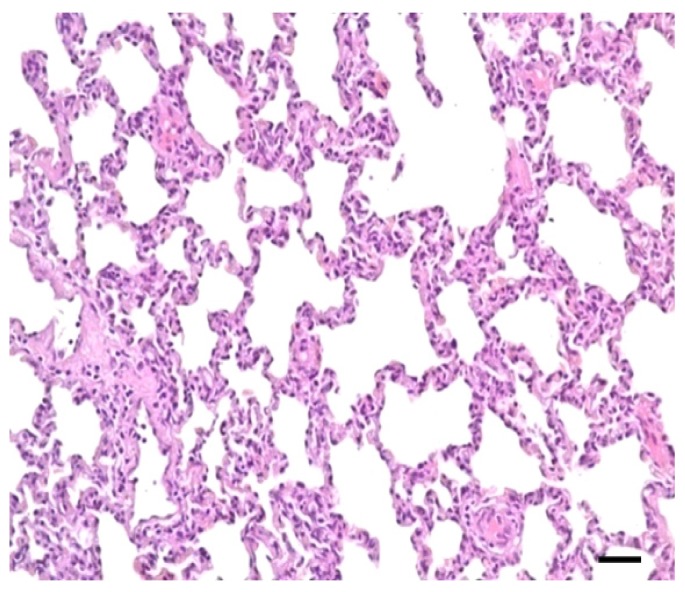
No lesions were observed in CG lung tissue (scale bar = 50 μm, 200×).

**Figure 3:**
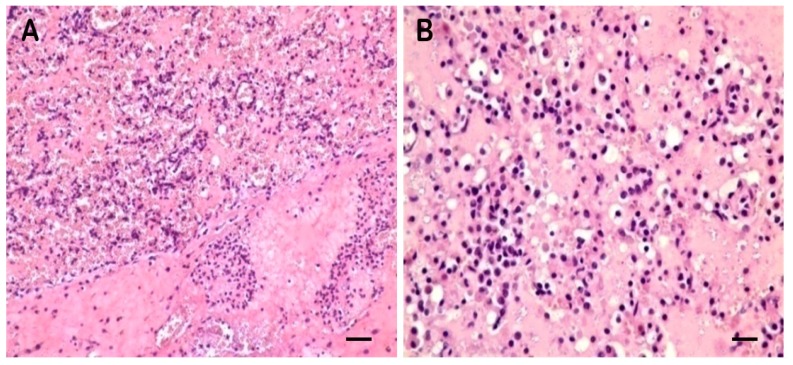
Alveolar cavities were filled with pink serum and red blood cells (**A**) (scale bar = 50 μm, 200×). and filled with serum, lymphocyte infiltration in the alveolar wall (**B**) (scale bar = 25 μm, 400×) in the lung of TG.

No lesions were observed in CG lung (Figures 1A and 2). Lung showed swelling, bleeding and fibrinous exudate sticking to the lung surface in TG (Figure 1B). The histopathologic changes were characterized by hemorrhage, lymphocyte infiltration, fibrinous exudation vascular thrombosis, necrotic focus and edema in TG (Figure 3).

### 2.2. Microarray Profiling

Expression profiling was conducted using a commercially available Agilent Porcine Genechip that included 43,603 probe sets. The transcriptome of the lung was determined. Expression was detected for 30,574 probes (70.12% of all probe sets) of the CG. A total of 31,957 probes (73.29% of all probe sets) were expressed in TG. When probe set intensities were normalized and filtered, there were still 26353 probes used to significantly identify DE genes. There were 11,929 genes identified as DE at the *p* ≤ 0.01 level by comparing the log2 (normalized signal) of the two groups using T-test analysis.

**Figure 4:**
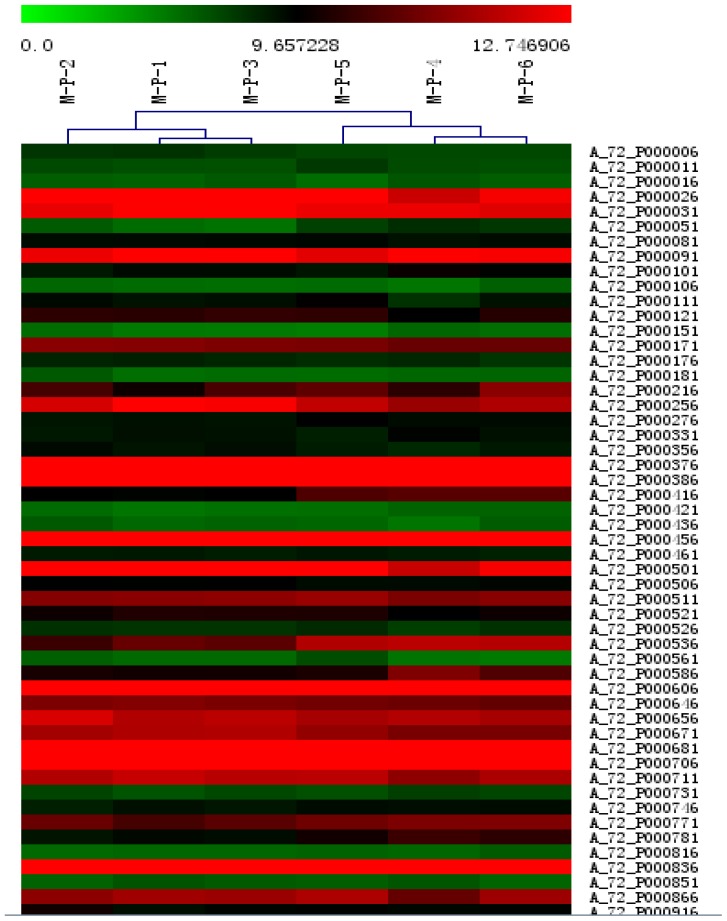
Hierarchical clustering analysis and clustering segmentation.

Hierarchical clustering was applied to the mean log-ratio of the replicated spots from the DE genes by the average linkage and using euclidean distance as the similarity metric (Figure 4). The expression profiles of samples were divided into two groups—one from the non-inoculated swine (M-P-1, M-P-2, M-P-3) and the other group from the inoculated swine (M-P-4, M-P-5, M-P-6).

**Figure 5:**
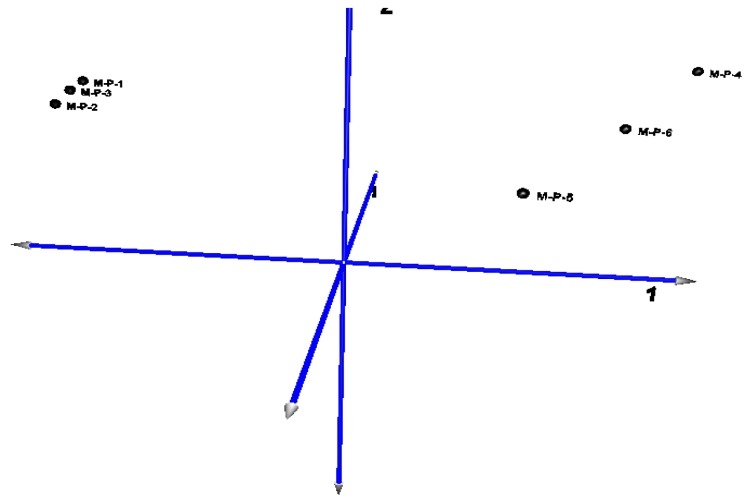
Three-dimensional map of principal component analysis (PCA) for mapping samples obtained from clustering segmentation.

The principal component map to three-dimensional space, also found that the distance of CG (samples M-P-1, M-P-2, M-P-3) is short, and the gene expression pattern is more consistent. Also the distance of TG (samples M-P-4, M-P-5, M-P-6) is relatively discrete because of the differences in the degree of lesion, and the gene expression pattern is similar (Figure 5).

The six samples were set as variables, the principal component analysis (PCA) of the co-expressed differentially genes (CG *vs* TG) showed the contribution rate of the first principal component which reached 96.95%, the first three principal components of the total contribution rate reach 99.754% (Table 1).

### 2.3. DE Genes Profiling

Of the 11,929 DE genes, 1188 were annotated as swine genes in the GenBank Database (DB). GO and KEGG pathway analyses of the 1188 DE gene lists were conducted using DAVID. There were 89 biological process (BP) categories (Table 2), 82 cellular components (Table 3), and 182 molecular functions (Table 4) were significantly affected by infection with APP (*p* = 0). The BP of the test for over-representation of specific GO terms among the affected genes related to the immune responses were at least 27 (Table 5). Furthermore, a number of BP related to metabolism were also identified.

A total of 513 genes were analyzed using gene set enrichment analysis (GSEA). Three hundred and thirty (64.3%) database genes correlated with TG, while the other 183 (35.7%) genes correlated with CG. One hundred and thirty pathways remained for further analysis after size filtering (2 ≤ sizes ≤ 20). Altogether, 102 pathways (Table 6) were enriched and up-regulated in the TG and down-regulated in the CG. One pathway (SSC04664) was significantly enriched at a false discovery rate <25%. Eight pathways (*i.e.*, SSC04664, SSC04930, SSC04914, SSC00140, SSC04621, SSC05221, SSC05218 and SSC03040) were significantly enriched at nominal *p* values of less than 1% and 5%.

Twenty-eight pathways (Table 7) were down-regulated in the TG but upregulated in the CG. Six pathways (*i.e.*, SSC05320, SSC04940, SSC05330, SSC04530, SSC04260 and SSC05412) were significant at a false discovery rate of <25%. Five pathways (*i.e.*, SSC05320, SSC04940, SSC05330, SSC04530 and SSC04260) were significantly enriched at a nominal p value of less than 1% and 5%.

**Figure 6:**
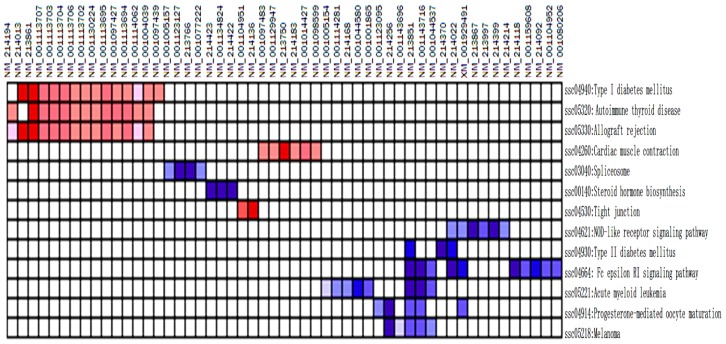
The heat map shows the clustered genes in the leading edge subsets. In the heat map, expression values are represented as colors, where the range of colors (red, pink, light blue, dark blue) represents the range of expression values (high, moderate, low, lowest) in the CG. This pattern is reversed in the TG.

Further analysis revealed that several immune response genes were induced by leading edge analysis for the 13 significant pathways (Figure 6). The genes included those encoding CCL2, GM-CSF, HLA-B associated transcript 1, IGF-1, IL-6, IL-8, IL-18, TNF, Hsp70s, Hsp70.2, Fc fragment of IgE, MAP2K1, PIK3R5, MAPK 14, STAT3 and STAT5B, among others. Many genes related with metabolism as well as ribosomal protein genes were also induced in the inflamed lung. These genes included adiponectin, the Saccharomyces pombe cell division cycle 25 homolog C, cytochrome P450 (3A29, 3A39 and 3A46), FYN oncogene related to SRC, FGR and YES (FYN), the phosphatase and tensin homolog PTEN, PDK, Snrpa, Syk, SPI1, and v-Ha-ras, c-Myc, avian, among others.

The repressed genes comprised those encoding members of the MHC, including (SLA- 2, SLA-3, SLA-6, SLA-8, SLA-DRA, SLA-DQA1, SLA-DRB1, SLA-DMB, SLA-DQA, SLA-DMA, SLA-DQB1), CD40 molecule, CD40, IL-12B, IL-2, myosin, MYH, MYH2, CACNB4, CACNA2D, CPE, and FXYD2, among others.

Genes were frequently induced in the TG included p101, MAP2K1, H-RAS, TNF, MAPK14 and IGF1, while the genes such as CD40, IL12B, IL-2, SLA-2, SLA-3, SLA-6, SLA-8, SLA-DRA, LA-DQA1, SLA-DRB1, SLA-DMB, SLA-DQA, SLA-DMA and SLA-DQB1 were frequently suppressed.

### 2.4. Verification of Gene Expression Pattern from Microarray Data Using Real-Time QRT-PCR

**Figure 7:**
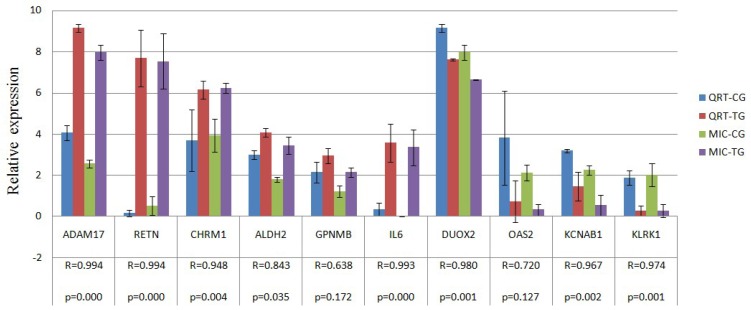
Validation of the microarray data by the real-time qRT-PCR analyses of ten representative genes. The x-axis represents the genes and the y-axis shows their relative expression levels (−ΔCt) values for quantitative real-time RT-PCR; Log (Sample signal, 10) for microarray. Three biological replicates were conducted for both assays. R represents the Pearson correlation coefficient. The significance of differences for gene expression between the CG and the TG was calculated using a two-tailed *T*-test.

Ten genes (*i.e.*, RETN, ADAM17, GPNMB, CHRM1, ALDH2, IL6, KLRK1, DUOX2, OAS2 and KCNAB1) were selected to confirm expression patterns using real-time qRT-PCR. The results indicate that the expression patterns of all the genes were consistent with the microarray data (*r* = 0.905 ± 0.125, Figure 7).

## 3. Discussion

In the present study, we revealed 11,929 DE genes using Agilent Whole Porcine Genome Oligo Microarrays (one-color platform) that contain 43,603 probes. There were 1188 genes annotated as swine genes in the GenBank Data Base (DB). GO term analysis identified that a total of 89 BP categories, 82 cellular components and 182 molecular functions were significantly affected and at least 27 BP categories were related to the host immune response.

The NOD-like receptor signaling pathway, Fc epsilon RI signaling pathway, acute myeloid leukemia, melanoma, progesterone-mediated oocyte maturation, spliceosome, type II diabetes mellitus and steroid hormone biosynthesis *etc.* were significantly enriched in inflamed lung. Five pathways as type I diabetes mellitus, autoimmune thyroid disease, allograft rejection, tight junction and cardiac muscle contraction were significantly enriched in non-inflamed lung. The NOD-like receptor signaling pathway is one of the most important pathways associated with microbial recognition and host defense [8,9]. The innate immune system comprises several classes of pattern recognition receptors, including Toll-like receptors (TLRs), NOD-like receptors (NLRs) and RIG-1-like receptors. Two NLRs, NOD1 and NOD2, sense the cytosolic presence of the peptidoglycan fragments, meso-DAP and muramyl dipeptide, respectively, and drive the activation of MAPK and the transcription factor NF-kappaB (NF-κB). A different set of NLRs induces caspase-1 activation through the assembly of large protein complexes named inflammasomes [9]. Inflammasomes are critical for generating mature proinflammatory cytokines in concert with Toll-like receptor signaling pathways [10]. Nod proteins fight off bacterial infections by stimulating proinflammatory signaling and cytokine networks and by inducing antimicrobial effectors, such as nitric oxide and antimicrobial peptides [11]. Fc epsilon RI-mediated signaling pathways in mast cells are initiated by the interaction of antigen with IgE bound to the extracellular domain of the alpha chain of the Fc epsilon RI [12–16]. The activation pathways are regulated by mast cells that release histamines and proteoglycans (especially heparin), lipid mediators such as leukotrienes (LTC4, LTD4 and LTE4) and prostaglandins (especially PDG2), and cytokines such as TNF-alpha, IL-4 and IL-5. These mediators and cytokines contribute to inflammatory response [14].

The pathways activated in infected lung tissues also include the acute myeloid leukemia pathway characterized by uncontrolled proliferation of clonal neoplastic cells and accumulation in the bone marrow of blasts with an impaired differentiation program [17–22], progesterone-mediated oocyte maturation pathway involved in endocrine system either insulin/IGF-1 or the steroid hormone progesterone regulation [23–25], steroid hormone biosynthesis pathway involved in lipid metabolism [26–29], type II diabetes mellitus involved in endocrine and metabolic diseases [30–33], spliceosome pathway involved in genetic information processing and transcription [34–36], and melanoma pathway involved in cancer arising from the malignant transformation of melanocytes [37–40].

The NOD-like receptor signaling pathway, Fc epsilon RI signaling pathway, acute myeloid leukemia pathway, progesterone-mediated oocyte maturation pathway were strongly linked to the MAPK signaling pathway, which are involved in environmental information processing and signal transduction [41–43]; the acute myeloid leukemia pathway progesterone-mediated oocyte maturation pathway, melanoma pathway were all strongly linked to the cell cycle pathway, which plays an important role in the regulation of cell growth and death [44–46]; and the NOD-like receptor signaling pathway, acute myeloid leukemia pathway, type II diabetes mellitus pathway, melanoma pathway were all directly or indirectly linked to the apoptosis pathway, which plays an important role in the regulation of apoptosis (programmed cell death) [47–49]. Hence, the pathways linked to the cell function regulation, including the MAPK signaling pathway, apoptosis and Cell cycle pathway, were also affected directly or indirectly by the process of the body’s resistance to infection.

Many cytokines as shown by leading edge analysis were activated at the site of inflammation, including IL-6, IL-8, IL-18, TNF, GM-CSF, CCL2, p101 protein, HLA-B associated transcript 1, Fc fragment of IgE, MAPK14, MAP2K1, IGF-1, STAT3 and STAT5B, *etc.* IL-6 is responsible for stimulating acute phase protein synthesis, as well as the production of neutrophils in the bone marrow. IL-8 is synthesized by macrophages, endothelial cells and epithelial cells as host defenses against severe infection [50,51]. It serves as a chemical signal that attracts neutrophils to the site of any inflammation. Significant increases in IL-8 and IL6- mRNA after infection with APP have previously been observed in lung lavage as well as lung tissue using northern blotting and *in situ* hybridization [52,53]. IL-18 plays multiple roles in chronic inflammation and in a number of infections and enhances both Th-1- and Th-2-mediated immune response [54]. IL-18 is able to induce IFN gamma, GM-CSF, TNF-α and IL-1 in immunocompetent cells to activate killing by lymphocytes and to up-regulate the expression of certain chemokine receptors. GM-CSF stimulates stem cells to produce granulocytes (neutrophils, eosinophils, and basophils) and monocytes. Monocytes exit the circulation and migrate into tissues, whereupon they mature into macrophages. Thus, these cells play a part in the immune-inflammatory cascade, by which activation of a small number of macrophages can rapidly lead to an increase in their number, a process crucial for fighting infection. CCL2 recruits monocytes, memory T cells and dendritic cells to the sites of tissue injury, infection, and inflammation [55,56]. TNF-α can promote inflammatory response by inducing the production of other proinflammatory cytokines at the vicinity of the infection [57], and increase the expression of endothelial surface HLA-B by activation of the nuclear transcription factor NF-κB [58,59]. P101 protein is a single regulatory subunit of the phosphoinositide 3-kinase gamma (PI3Kα), which plays a crucial role in inflammatory and allergic processes [60,61], including neutrophil chemotaxis, mast cell degranulation, and cardiac function [62,63].

Genes involved in a variety of cellular function, including proliferation, differentiation, growth arrest or apoptosis of normal cells were affected including those encoding HLA-B associated transcript 1 [64,65], Fc fragment of IgE [66,67], MAPK14 [68], MAP2K1 [69], H-ras [70,71], IGF-1 [72], STAT3 and STAT5B [73]. Activations of all these genes can stimulate stem cells to produce granulocytes (neutrophils, eosinophils, and basophils) and monocytes, and also induce neutrophils and macrophages to phagocytose bacterial and foreign antigens.

Immunomodulatory cytokines were significantly suppressed at the site of inflammation. In this study, genes encoding IL2, IL12B, CD40, members of the MHC (SLA-2, SLA-3, SLA-6, SLA-8, SLA-DRB1, SLA-DMB, SLA-DQA, SLA-DMA and SLA-DQB1), as well as SLA-DRA and SLA-DQA1 in a previous study [74], were significantly down-regulated at the site of inflammation.

IL-2 is a type of cytokine immune system signaling molecule which is a leukocytotrophic hormone made in response to microbial infection that can identify the difference between self and non-self [75,76]. When environmental substances (molecules or microbes) gain access to the body, these substances (termed antigens) are recognized as foreign by antigen receptors that are expressed on the surface of lymphocytes. MHC can present antigenic peptides to T lymphocytes, which are responsible for a specific immune response that can destroy the pathogen producing those antigens [77]. CD40 is a co-stimulatory protein found on antigen presenting cells (APC) and is essential in mediating a broad variety of immune and inflammatory responses including T cell-dependent immunoglobulin class switching, memory B cell development, and germinal center formation [78]. The macrophage can express more CD40 and TNF receptors on its surface, which can increase the level of activation culminating in the induction of potent microbicidal substances in the macrophage; these include reactive oxygen species and nitric oxide, leading to the destruction of the ingested microbe [79–82]. IL-12 is an essential inducer of Th1 cell development, and has an important role in sustaining a sufficient number of memory/effector Th1 cells to mediate long-term protection against an intracellular pathogen [83]. The suppression of these immunomodulatory cytokines leads to a decrease in antigenic peptides presented to T lymphocytes by APC via the MHC, as well as to alleviate immune response injury induced by infection at the site of inflammation.

Many genes encoding metabolism as well as ribosomal proteins were affected at the site of inflammation. Genes related to metabolism and ribosomal proteins synthesis were induced in the inflamed lung, including adiponectin, cell division cycle 25 homolog C, cytochrome P450 (CYP 3A29, CYP 3A39 and CYP 3A46), FYN, PTEN, PDK, Snrpa, Syk and SPI1, among others. The repressed genes comprised those encoding MYH1, MYH2, tropomyosin (alpha, beta), troponin I type 3 (cardiac), CACNB4, CACNA2D1, CPE and FXYD2; while the CYP2E1 and the CYP3A29 were known to be down-regulated during inflammation in another study [84].

SOCS3 and CISH, both found to be up-regulated in the present study, are members of the suppressor of cytokine signaling (SOCS) family of proteins whose members regulate protein turnover by targeting proteins for degradation [42]. Expression of the members of the SOCS family is induced by cytokines such as IL-6 and IL-10, both found to be up-regulated in this study; both function as negative feed- back regulators of cytokine signaling [85,86]. The statistically significant increase in mRNA coding for the anti-inflammatory cytokine IL-10, found in inflamed areas of the lung, is probably due to the function of IL-10 in counteracting the host mediated tissue damage caused by proinflammatory and chemotactic cytokines [87]. The lower expression levels observed for genes encoding ribosomal proteins could be due to a general downregulation of ribosomal biogenesis in the necrotic areas of the lung. Previous studies have shown that 41 out of 54 genes encoding ribosomal proteins were down-regulated in Pseudomonas aeruginosa after treatment with H_2_O_2_ induced oxidative stress [88].

As described above, we found that: (1) A total of 89 biological process categories, 82 cellular components and 182 molecular functions were significantly affected, and more than 27 biological process were involved in the host immune response; (2) At the site of inflammation, 13 pathways associated with host responses were affected significantly; many proinflammatory-inflammatory cytokines were activated and several immunomodulatory cytokines were suppressed at the gene expression level reflecting the complex machinery at work during an infection; (3) Many genes which were involved in a variety of cellular functions-proliferation, differentiation, growth arrest or apoptosis of normal cells that activated, could stimulate stem cells to produce granulocyte (neutrophil, eosinophil, and basophil) and monocyte. All changes of the genes and pathways which induced or repressed expression, not only led to decrease in antigenic peptides presented to T lymphocytes by APC via the MHC and alleviated immune response injury induced by infection, but also stimulated stem cells to produce granulocyte (neutrophil, eosinophil, and basophil ) and monocyte, and promote neutrophil and macrophages to phagocytose bacterial and foreign antigen at the site of inflammation. Additional work including more animals and time points is clearly needed to further delineate the host response to APP infection and will contribute to a more detailed description of the dynamics of host responses in general.

## 4. Experimental Section

### 4.1. Animals, Bacterial Inoculation and Samples

All animal procedures were performed according to protocols approved by the Biological Studies Animal Care and Use Committee of Sichuan Province, China. Twenty 12-week-old male castrated Danish Landrace/Yorkshire/Duroc crossbred swine from a healthy herd free from APP were divided equally into a control group (CG) and the treatment group (TG). APP serotype I (Strain provided by the Animal Biotechnology Center, Laboratory of Animal Disease and Human Health, Sichuan Agricultural University) was cultivated overnight at 37 °C in air on trypticase soy broth (TSB) (Hangwei, Hangzhou, China). Bacterial counts of the suspensions were performed at the same time as the start of the inoculation. The inoculation was performed by holding the pigs (1–10) from the TG in an upright sitting position and spraying 0.25mL diluent containing (3.5–4) × 10^7^ CFU/mL APP per kilogram weight into the nostrils during inspiration. Swine from the CG (swines 11–20) were inoculated with physiological saline (0.9% *wt*/*vol* NaCl) by the same means. In the TG, lung tissue was collected from three swine (swines 1, 2 and 3) after abattage and used for total RNA extraction and pathological analysis. Another three swine (swines 11, 12 and 13) from the CG were sacrificed 48 h post-inoculation and their lung tissues were collected. The remaining swine were used for other trials.

### 4.2. Microarray Hybridizations and Data Analysis

Total RNA was extracted from tissues using Trizol reagent (Invitrogen, Carlsbad, CA, USA). RNA was purified and DNase treated using the RNeasy QIAGEN RNeasy^®^ Mini Kit. cDNA was synthesized from 2 μg of total-RNA using the direct cDNA Labeling System. Aminoallyl-cRNA was synthesized from cDNA using the Superscript Indirect cDNA Labeling System. The cRNA was purified and DNase treated using RNeasy QIAGEN RNeasy^®^ Mini Kit. RNA integrity was confirmed with a bioanalyzer (model 2100; Agilent Technologies, Palo Alto, CA, USA) according to the manufacturer’s protocol. Labeling and hybridization of the cRNA was performed with Agilent Whole Porcine Genome Oligo (4 × 44 K) Microarrays (one-color platform) at the National Engineering Center for Biochip at Shanghai, according to the manufacturer’s protocols. The slides were scanned and analyzed using the histogram method with default settings in an Agilent G2565AA and Agilent G2565BA Microarray Scanner System with SureScan Technology. The array data were submitted to GEO [89].

Comparisons between the CG and TG were carried out using three biological replicates for each group. CG samples and TG samples were used for microarray analysis. The six Microarray data were normalized using the quantile normalization method [90] with WebarrayDB (http://www.webarraydb.org/webarray/) [91] and were filtered and assessed by the MIDAW online analysis program (http://www.webarraydb.org/webarray/) [92] using the method of weighted K-nearest neighbor [93]. *T*-tests and hierarchical cluster analyses of the significantly differentially expressed (DE) genes (clustering method: complete linkage; similarity measure: Pearson product momentum correlation; ordering function: average value) for microarray data were carried out by a MultiExperiment Viewer (MeV) software package (Version 4.5, Dana-Farber Cancer Institute, Boston, MA, USA, 2009) [94].

Tests for statistical significance (*p* < 0.05), overrepresentation of Gene ontology (GO) terms [74,95], and pathway in Kyoto Encyclopedia of Genes and Genomes (KEGG) DB [10,96] (http://www.genome.jp/kegg/) both induced and repressed genes were conducted using the ErmineJ [97] and the Database for Annotation, Visualization and Integrated Discovery (DAVID) Online platform (http://david.abcc.ncifcrf.gov/) with a threshold of a minimum three genes annotated at each node. The leading edge analysis for the pathway of differential expression in microarray data with a threshold of a minimum of two genes and maximum of 20 genes annotated at each node was conducted using the GSEA V2.06 package [98,99]. More detailed descriptions of the microarray experiments are available at the NCBI Gene Expression Omnibus [100–102].

### 4.3. Real-Time QRT-PCR

In order to confirm the reliability the expression profile in the microarray analyses, the expression level 10 gene (six up-regulated and four down-regulated) were performed by real-time qRT-PCR. Sequences for primers were obtained from Genbank and NCBI. Primers were designed using Primer 5 and synthesized at Invitrogen (Shanghai, China) (Table 1). Extracted RNA was converted into cDNA by reverse transcription of 1 μL total RNA using SYBR® PrimeScriptTM RT-PCR Kit (TaKaRa, Japan) according to the manufacturer’s protocol and then cDNA was stored at −20 °C until use. Quantitative PCR was performed in a 25 μL reaction volume (2 μL cDNA, 12.5 μL of SYBR^®^ Premix Ex TaqTM (2×) TaKaRa, Japan), 0.5 μL of 10 μM upstream and downstream primers respectively, and added ddH_2_O to 25 μL) on the BIO-RAD IQ5 System (BIO-RAD, Hercules, CA, USA). Real-time PCR conditions were as follows: 30 s at 95.0 °C, 40 cycles of denaturation at 95 °C for 5 s followed by 30 s annealing and elongation at 51.2–60 °C (Table 8). Efficiency of primer pairs is reported in Table 1. Melting curves were obtained at the end of each run to confirm a single PCR product. All samples were run in triplicate. Non-template controls were included in each run to exclude contamination and nonspecific amplification. Expression levels of samples were normalised by using a normalisation factor calculated by the program geNorm. This normalisation factor was calculated based on RT-qPCR results for three selected reference genes, ACTB, TOP2B and TBP.

This allowed quantification of the target gene in one sample relative to that in another (the calibrator) using the “2^−ΔΔCt^ method” of calculating fold changes in gene expression [103]. Correlation analysis between qRT-PCR and microarray was conducted.

## 5. Conclusions

We have generated reliable mRNA transcriptomes of swine lung tissues from APP-infected and negative control pigs. We have identified a set of differentially expressed (DE) genes in our current case-control study, and a functional enrichment analysis indicated that these DE genes mainly related to “host immune response” and “host response”. In addition, we also found that, in the APP-infected lung tissues, many proinflammatory-inflammatory cytokines were activated and involved in the regulation of the host defense response at the site of inflammation, while the cytokines involved in regulation of the host immune response were suppressed. The current study provides data that can be used in future studies to decipher the molecular mechanism of the systematic influences from porcine pleuropneumonia. Our findings will also help promote the further development of therapy for porcine pleuropneumonia.

## Figures and Tables

**Table 1:** Eigenvalues and contribution ratio of principal component analysis (PCA) for differential expression genes.

Principal component	Eigenvalues	Contribution ratio
1	50.17	96.95%
2	1.339	2.59%
3	0.111	0.21%
4	0.088	0.17%
5	0.027	0.05%
6	0.013	0.02%

**Table 2:** The significant gene ontology biological processes in pigs.

Name	Description	Probe	Genes
[GO:0050896]	response to stimulus	96	96
[GO:0051179]	Localization	92	92
[GO:0006810]	Transport	88	88
[GO:0006807]	nitrogen compound metabolic process	84	84
[GO:0019222]	regulation of metabolic process	67	67
[GO:0002376]	immune system process	56	56
[GO:0055114]	oxidation reduction	53	53
[GO:0006955]	immune response	53	53
[GO:0032501]	multicellular organismal process	52	52
[GO:0006950]	response to stress	51	51
[GO:0009056]	catabolic process	50	50
[GO:0032502]	developmental process	38	38
[GO:0065008]	regulation of biological quality	35	35
[GO:0007275]	multicellular organismal development	35	35
[GO:0022610]	biological adhesion	29	29
[GO:0048518]	positive regulation of biological process	28	28
[GO:0016043]	cellular component organization	27	27
[GO:0009605]	response to external stimulus	25	25
[GO:0048856]	anatomical structure development	22	22
[GO:0008219]	cell death	22	22
[GO:0048519]	negative regulation of biological process	21	21
[GO:0033036]	macromolecule localization	21	21
[GO:0048523]	negative regulation of cellular process	19	19
[GO:0048522]	positive regulation of cellular process	19	19
[GO:0044281]	small molecule metabolic process	18	18
[GO:0048869]	cellular developmental process	17	17
[GO:0042221]	response to chemical stimulus	17	17
[GO:0006066]	alcohol metabolic process	17	17
[GO:0006996]	organelle organization	16	16
[GO:0051641]	cellular localization	15	15
[GO:0019882]	antigen processing and presentation	15	15
[GO:0008104]	protein localization	15	15
[GO:0048583]	regulation of response to stimulus	13	13
[GO:0042592]	homeostatic process	12	12
[GO:0032879]	regulation of localization	12	12
[GO:0007049]	cell cycle	12	12
[GO:0048584]	positive regulation of response to stimulus	11	11
[GO:0009893]	positive regulation of metabolic process	11	11
[GO:0002682]	regulation of immune system process	11	11
[GO:0051716]	cellular response to stimulus	10	10
[GO:0019725]	cellular homeostasis	10	10
[GO:0016192]	vesicle-mediated transport	10	10
[GO:0009653]	anatomical structure morphogenesis	10	10
[GO:0002252]	immune effector process	10	10
[GO:0051239]	regulation of multicellular organismal process	9	9
[GO:0048731]	system development	9	9
[GO:0065009]	regulation of molecular function	8	8
[GO:0051704]	multi-organism process	8	8
[GO:0051128]	regulation of cellular component organization	8	8
[GO:0050778]	positive regulation of immune response	8	8
[GO:0044085]	cellular component biogenesis	8	8
[GO:0007610]	behavior	8	8
[GO:0003008]	system process	8	8
[GO:0051301]	cell division	7	7
[GO:0030029]	actin filament-based process	7	7
[GO:0022607]	cellular component assembly	7	7
[GO:0009607]	response to biotic stimulus	7	7
[GO:0000003]	reproduction	7	7
[GO:0055085]	transmembrane transport	6	6
[GO:0051707]	response to other organism	6	6
[GO:0051129]	negative regulation of cellular component organization	6	6
[GO:0050878]	regulation of body fluid levels	6	6
[GO:0050793]	regulation of developmental process	6	6
[GO:0023052]	signaling	6	6
[GO:0022414]	reproductive process	6	6
[GO:0016044]	cellular membrane organization	6	6
[GO:0048646]	anatomical structure formation involved in morphogenesis	5	5
[GO:0040011]	locomotion	5	5
[GO:0019953]	sexual reproduction	5	5
[GO:0019637]	organophosphate metabolic process	5	5
[GO:0010817]	regulation of hormone levels	5	5
[GO:0009892]	negative regulation of metabolic process	5	5
[GO:0060348]	bone development	4	4
[GO:0044087]	regulation of cellular component biogenesis	4	4
[GO:0043933]	macromolecular complex subunit organization	4	4
[GO:0042330]	taxis	4	4
[GO:0040008]	regulation of growth	4	4
[GO:0022402]	cell cycle process	4	4
[GO:0070271]	protein complex biogenesis	3	3
[GO:0048609]	reproductive process in a multicellular organism	3	3
[GO:0046903]	secretion	3	3
[GO:0040012]	regulation of locomotion	3	3
[GO:0034621]	cellular macromolecular complex subunit organization	3	3
[GO:0019748]	secondary metabolic process	3	3
[GO:0010605]	negative regulation of macromolecule metabolic process	3	3
[GO:0009719]	response to endogenous stimulus	3	3
[GO:0009628]	response to abiotic stimulus	3	3
[GO:0007017]	microtubule-based process	3	3
[GO:0002520]	immune system development	3	3

**Table 3:** The significant gene ontology cellular components in pigs.

Name	Description	Probe	Genes
[GO:0005886]	plasma membrane	98	98
[GO:0005634]	nucleus	86	86
[GO:0032991]	macromolecular complex	86	86
[GO:0044422]	organelle part	84	84
[GO:0043234]	protein complex	62	62
[GO:0043228]	non-membrane-bounded organelle	46	46
[GO:0044421]	extracellular region part	45	45
[GO:0044459]	plasma membrane part	44	44
[GO:0031090]	organelle membrane	41	41
[GO:0005739]	mitochondrion	37	37
[GO:0005783]	endoplasmic reticulum	35	35
[GO:0005794]	Golgi apparatus	33	33
[GO:0005615]	extracellular space	29	29
[GO:0012505]	endomembrane system	25	25
[GO:0044429]	mitochondrial part	23	23
[GO:0016023]	cytoplasmic membrane-bounded vesicle	23	23
[GO:0005856]	cytoskeleton	23	23
[GO:0031974]	membrane-enclosed lumen	22	22
[GO:0031975]	envelope	21	21
[GO:0005840]	ribosome	19	19
[GO:0044428]	nuclear part	18	18
[GO:0005578]	proteinaceous extracellular matrix	17	17
[GO:0044430]	cytoskeletal part	15	15
[GO:0071212]	subsynaptic reticulum	15	15
[GO:0005740]	mitochondrial envelope	15	15
[GO:0005829]	cytosol	14	14
[GO:0031966]	mitochondrial membrane	14	14
[GO:0019898]	extrinsic to membrane	14	14
[GO:0042611]	MHC protein complex	13	13
[GO:0048770]	pigment granule	12	12
[GO:0044431]	Golgi apparatus part	12	12
[GO:0005773]	vacuole	11	11
[GO:0005764]	lysosome	10	10
[GO:0044432]	endoplasmic reticulum part	10	10
[GO:0005792]	microsome	9	9
[GO:0009898]	internal side of plasma membrane	9	9
[GO:0005743]	mitochondrial inner membrane	9	9
[GO:0005887]	integral to plasma membrane	8	8
[GO:0005789]	endoplasmic reticulum membrane	8	8
[GO:0005768]	endosome	8	8
[GO:0005759]	mitochondrial matrix	8	8
[GO:0005654]	nucleoplasm	8	8
[GO:0015630]	microtubule cytoskeleton	8	8
[GO:0030054]	cell junction	7	7
[GO:0031300]	intrinsic to organelle membrane	7	7
[GO:0042613]	MHC class II protein complex	7	7
[GO:0044451]	nucleoplasm part	7	7
[GO:0031301]	integral to organelle membrane	6	6
[GO:0005635]	nuclear envelope	6	6
[GO:0042612]	MHC class I protein complex	6	6
[GO:0015629]	actin cytoskeleton	6	6
[GO:0016469]	proton-transporting two-sector ATPase complex	6	6
[GO:0031225]	anchored to membrane	5	5
[GO:0031968]	organelle outer membrane	5	5
[GO:0031965]	nuclear membrane	5	5
[GO:0043235]	receptor complex	5	5
[GO:0033279]	ribosomal subunit	5	5
[GO:0005819]	spindle	4	4
[GO:0030173]	integral to Golgi membrane	4	4
[GO:0048471]	perinuclear region of cytoplasm	4	4
[GO:0005911]	cell-cell junction	4	4
[GO:0043292]	contractile fiber	4	4
[GO:0000502]	proteasome complex	4	4
[GO:0030135]	coated vesicle	4	4
[GO:0016459]	myosin complex	4	4
[GO:0005874]	microtubule	4	4
[GO:0042995]	cell projection	3	3
[GO:0045259]	proton-transporting ATP synthase complex	3	3
[GO:0044420]	extracellular matrix part	3	3
[GO:0015935]	small ribosomal subunit	3	3
[GO:0005777]	peroxisome	3	3
[GO:0034702]	ion channel complex	3	3
[GO:0005901]	caveola	3	3
[GO:0045202]	synapse	3	3
[GO:0031227]	intrinsic to endoplasmic reticulum membrane	3	3
[GO:0016323]	basolateral plasma membrane	3	3
[GO:0005681]	spliceosomal complex	3	3
[GO:0030141]	secretory granule	3	3
[GO:0005667]	transcription factor complex	3	3
[GO:0033176]	proton-transporting V-type ATPase complex	3	3
[GO:0033177]	proton-transporting two-sector ATPase complex, proton-transporting domain	3	3
[GO:0005730]	nucleolus	3	3

**Table 4:** The significant gene ontology molecular functions in pigs.

Name	Description	Probe	Genes
[GO:0017076]	purine nucleotide binding	91	91
[GO:0003676]	nucleic acid binding	88	88
[GO:0032555]	purine ribonucleotide binding	81	81
[GO:0004872]	receptor activity	75	75
[GO:0004690]	cyclic nucleotide-dependent protein kinase activity	68	68
[GO:0004691]	cAMP-dependent protein kinase activity	67	67
[GO:0016491]	oxidoreductase activity	67	67
[GO:0008270]	zinc ion binding	64	64
[GO:0030554]	adenyl nucleotide binding	63	63
[GO:0005102]	receptor binding	57	57
[GO:0032559]	adenyl ribonucleotide binding	53	53
[GO:0005215]	transporter activity	52	52
[GO:0008233]	peptidase activity	46	46
[GO:0070011]	peptidase activity, acting on l-amino acid peptides	43	43
[GO:0003677]	DNA binding	43	43
[GO:0004888]	transmembrane receptor activity	40	40
[GO:0005509]	calcium ion binding	39	39
[GO:0022892]	substrate-specific transporter activity	39	39
[GO:0004687]	myosin light chain kinase activity	38	38
[GO:0030528]	transcription regulator activity	37	37
[GO:0022857]	transmembrane transporter activity	36	36
[GO:0022891]	substrate-specific transmembrane transporter activity	35	35
[GO:0030234]	enzyme regulator activity	35	35
[GO:0005506]	iron ion binding	33	33
[GO:0004175]	endopeptidase activity	33	33
[GO:0003700]	transcription factor activity	32	32
[GO:0015075]	ion transmembrane transporter activity	30	30
[GO:0019001]	guanyl nucleotide binding	28	28
[GO:0005198]	structural molecule activity	28	28
[GO:0004857]	enzyme inhibitor activity	26	26
[GO:0016788]	hydrolase activity, acting on ester bonds	26	26
[GO:0008324]	cation transmembrane transporter activity	25	25
[GO:0009055]	electron carrier activity	24	24
[GO:0004930]	G-protein coupled receptor activity	24	24
[GO:0003723]	RNA binding	23	23
[GO:0005126]	cytokine receptor binding	22	22
[GO:0016874]	ligase activity	22	22
[GO:0048037]	cofactor binding	20	20
[GO:0016817]	hydrolase activity, acting on acid anhydrides	19	19
[GO:0003735]	structural constituent of ribosome	19	19
[GO:0005125]	cytokine activity	18	18
[GO:0030414]	peptidase inhibitor activity	18	18
[GO:0050662]	coenzyme binding	17	17
[GO:0016757]	transferase activity, transferring glycosyl groups	17	17
[GO:0030246]	carbohydrate binding	17	17
[GO:0008092]	cytoskeletal protein binding	16	16
[GO:0022890]	inorganic cation transmembrane transporter activity	16	16
[GO:0016746]	transferase activity, transferring acyl groups	16	16
[GO:0016879]	ligase activity, forming carbon-nitrogen bonds	16	16
[GO:0000287]	magnesium ion binding	15	15
[GO:0008237]	metallopeptidase activity	15	15
[GO:0016614]	oxidoreductase activity, acting on CH–OH group of donors	15	15
[GO:0022804]	active transmembrane transporter activity	15	15
[GO:0019955]	cytokine binding	14	14
[GO:0017171]	serine hydrolase activity	14	14
[GO:0046906]	tetrapyrrole binding	14	14
[GO:0016616]	oxidoreductase activity, acting on the CH–OH group of donors, NAD or NADP as acceptor	14	14
[GO:0016747]	transferase activity, transferring acyl groups other than amino-acyl groups	14	14
[GO:0008528]	peptide receptor activity, G-protein coupled	14	14
[GO:0003779]	actin binding	14	14
[GO:0004252]	serine-type endopeptidase activity	13	13
[GO:0016705]	oxidoreductase activity, acting on paired donors, with incorporation or reduction of molecular oxygen	13	13
[GO:0004497]	monooxygenase activity	12	12
[GO:0042578]	phosphoric ester hydrolase activity	12	12
[GO:0008234]	cysteine-type peptidase activity	11	11
[GO:0046873]	metal ion transmembrane transporter activity	11	11
[GO:0008289]	lipid binding	11	11
[GO:0016791]	phosphatase activity	11	11
[GO:0050660]	FAD binding	10	10
[GO:0015078]	hydrogen ion transmembrane transporter activity	10	10
[GO:0016758]	transferase activity, transferring hexosyl groups	10	10
[GO:0004867]	serine-type endopeptidase inhibitor activity	10	10
[GO:0004428]	inositol or phosphatidylinositol kinase activity	10	10
[GO:0016798]	hydrolase activity, acting on glycosyl bonds	10	10
[GO:0005516]	calmodulin binding	9	9
[GO:0016810]	hydrolase activity, acting on carbon-nitrogen (but not peptide) bonds	9	9
[GO:0042623]	ATPase activity, coupled	9	9
[GO:0001871]	pattern binding	9	9
[GO:0016776]	phosphotransferase activity, phosphate group as acceptor	9	9
[GO:0005179]	hormone activity	9	9
[GO:0070851]	growth factor receptor binding	9	9
[GO:0004197]	cysteine-type endopeptidase activity	9	9
[GO:0005057]	receptor signaling protein activity	9	9
[GO:0004950]	chemokine receptor activity	9	9
[GO:0004222]	metalloendopeptidase activity	9	9
[GO:0043565]	sequence-specific DNA binding	9	9
[GO:0005216]	ion channel activity	8	8
[GO:0016853]	isomerase activity	8	8
[GO:0000826]	inositol pyrophosphate synthase activity	8	8
[GO:0019842]	vitamin binding	8	8
[GO:0005539]	glycosaminoglycan binding	8	8
[GO:0005529]	sugar binding	8	8
[GO:0005066]	transmembrane receptor protein tyrosine kinase signaling protein activity	8	8
[GO:0020037]	heme binding	8	8
[GO:0004356]	glutamate-ammonia ligase activity	8	8
[GO:0005507]	copper ion binding	7	7
[GO:0016209]	antioxidant activity	7	7
[GO:0008238]	exopeptidase activity	7	7
[GO:0008009]	chemokine activity	7	7
[GO:0016860]	intramolecular oxidoreductase activity	7	7
[GO:0004721]	phosphoprotein phosphatase activity	7	7
[GO:0015291]	secondary active transmembrane transporter activity	7	7
[GO:0016563]	transcription activator activity	6	6
[GO:0005244]	voltage-gated ion channel activity	6	6
[GO:0008201]	heparin binding	6	6
[GO:0031420]	alkali metal ion binding	6	6
[GO:0046983]	protein dimerization activity	6	6
[GO:0015082]	di-, tri-valent inorganic cation transmembrane transporter activity	6	6
[GO:0004312]	fatty-acid synthase activity	6	6
[GO:0042802]	identical protein binding	6	6
[GO:0016684]	oxidoreductase activity, acting on peroxide as acceptor	6	6
[GO:0016829]	lyase activity	5	5
[GO:0008047]	enzyme activator activity	5	5
[GO:0003924]	GTPase activity	5	5
[GO:0004091]	carboxylesterase activity	5	5
[GO:0015399]	primary active transmembrane transporter activity	5	5
[GO:0005261]	cation channel activity	5	5
[GO:0019904]	protein domain specific binding	5	5
[GO:0004694]	eukaryotic translation initiation factor 2alpha kinase activity	5	5
[GO:0016627]	oxidoreductase activity, acting on the CH–CH group of donors	5	5
[GO:0031406]	carboxylic acid binding	5	5
[GO:0042803]	protein homodimerization activity	5	5
[GO:0004518]	nuclease activity	5	5
[GO:0019899]	enzyme binding	5	5
[GO:0003774]	motor activity	5	5
[GO:0008430]	selenium binding	5	5
[GO:0004725]	protein tyrosine phosphatase activity	5	5
[GO:0015293]	symporter activity	5	5
[GO:0004713]	protein tyrosine kinase activity	5	5
[GO:0046915]	transition metal ion transmembrane transporter activity	4	4
[GO:0008135]	translation factor activity, nucleic acid binding	4	4
[GO:0008134]	transcription factor binding	4	4
[GO:0010857]	calcium-dependent protein kinase activity	4	4
[GO:0050661]	NADP or NADPH binding	4	4
[GO:0060589]	nucleoside-triphosphatase regulator activity	4	4
[GO:0008373]	sialyltransferase activity	4	4
[GO:0004896]	cytokine receptor activity	4	4
[GO:0008083]	growth factor activity	4	4
[GO:0016709]	oxidoreductase activity, acting on paired donors, with incorporation or reduction of molecular oxygen, NADH or NADPH as one donor, and incorporation of one atom of oxygen	4	4
[GO:0004576]	oligosaccharyl transferase activity	4	4
[GO:0008509]	anion transmembrane transporter activity	4	4
[GO:0051540]	metal cluster binding	4	4
[GO:0004177]	aminopeptidase activity	4	4
[GO:0016765]	transferase activity, transferring alkyl or aryl (other than methyl) groups	4	4
[GO:0015929]	hexosaminidase activity	4	4
[GO:0016741]	transferase activity, transferring one-carbon groups	4	4
[GO:0004129]	cytochrome-c oxidase activity	4	4
[GO:0004521]	endoribonuclease activity	4	4
[GO:0051287]	NAD or NADH binding	3	3
[GO:0005385]	zinc ion transmembrane transporter activity	3	3
[GO:0004774]	succinate-CoA ligase activity	3	3
[GO:0004090]	carbonyl reductase (NADPH) activity	3	3
[GO:0005249]	voltage-gated potassium channel activity	3	3
[GO:0008757]	S-adenosylmethionine-dependent methyltransferase activity	3	3
[GO:0005160]	transforming growth factor beta receptor binding	3	3
[GO:0008235]	metalloexopeptidase activity	3	3
[GO:0005275]	amine transmembrane transporter activity	3	3
[GO:0019840]	isoprenoid binding	3	3
[GO:0019838]	growth factor binding	3	3
[GO:0005128]	erythropoietin receptor binding	3	3
[GO:0016801]	hydrolase activity, acting on ether bonds	3	3
[GO:0016701]	oxidoreductase activity, acting on single donors with incorporation of molecular oxygen	3	3
[GO:0003712]	transcription cofactor activity	3	3
[GO:0015144]	carbohydrate transmembrane transporter activity	3	3
[GO:0003995]	acyl-CoA dehydrogenase activity	3	3
[GO:0004869]	cysteine-type endopeptidase inhibitor activity	3	3
[GO:0031402]	sodium ion binding	3	3
[GO:0019888]	protein phosphatase regulator activity	3	3
[GO:0016651]	oxidoreductase activity, acting on NADH or NADPH	3	3
[GO:0004180]	carboxypeptidase activity	3	3
[GO:0030695]	GTPase regulator activity	3	3
[GO:0010181]	FMN binding	3	3
[GO:0003743]	translation initiation factor activity	3	3
[GO:0016861]	intramolecular oxidoreductase activity, interconverting aldoses and ketoses	3	3
[GO:0004522]	pancreatic ribonuclease activity	3	3
[GO:0015294]	solute:cation symporter activity	3	3
[GO:0016790]	thiolester hydrolase activity	3	3
[GO:0008026]	ATP-dependent helicase activity	3	3
[GO:0030145]	manganese ion binding	3	3
[GO:0008417]	fucosyltransferase activity	3	3
[GO:0008194]	UDP-glycosyltransferase activity	3	3
[GO:0008199]	ferric iron binding	3	3

**Table 5:** Significant GO terms related to the immune responses caused by infection with APP.

NO	GO term	Biological process	Number P_ermineJ_	
1	GO:0050896	response to stimulus	96	0
2	GO:0051179	Localization	92	0
3	GO:0002376	immune system process	56	0
4	GO:0006955	immune response	53	0
5	GO:0055114	oxidation reduction	53	0
1	GO:0050896	response to stimulus	96	0
6	GO:0006950	response to stress	51	0
7	GO:0022610	biological adhesion	29	0
8	GO:0009605	response to external stimulus	25	0
9	GO:0008219	cell death	22	0
10	GO:0033036	macromolecule localization	21	0
11	GO:0042221	response to chemical stimulus	17	0
12	GO:0019882	antigen processing and presentation	15	0
13	GO:0048583	regulation of response to stimulus	13	0
14	GO:0032879	regulation of localization	12	0
15	GO:0042592	homeostatic process	12	0
16	GO:0048584	positive regulation of response to stimulus	11	0
17	GO:0002682	regulation of immune system process	11	0
18	GO:0051716	cellular response to stimulus	10	0
19	GO:0002252	immune effector process	10	0
20	GO:0050778	positive regulation of immune response	8	0
21	GO:0009607	response to biotic stimulus	7	0
22	GO:0023052	signaling	6	0
23	GO:0040011	locomotion	5	0
24	GO:0042330	taxis	4	0
25	GO:0002520	immune system development	3	0
26	GO:0009628	response to abiotic stimulus	3	0
27	GO:0009719	response to endogenous stimulus	3	0

**Table 6:** Gene sets enriched in phenotype treatment group (TG).

NO.	Pathway	Size	ES	NES	NOM *p*-val	FDR *q*-val	FWER *p*-val	Rank at max	Leading edge
1	ssc04664: Fc epsilon RI signaling pathway	13	−0.74	−1.83	0.001	0.084	0.064	89	Tags = 77%, list = 17%, signal = 91%
2	ssc04930: Type II diabetes mellitus	5	−0.82	−1.62	0.017	0.715	0.685	42	Tags = 60%, list = 8%, signal = 65%
3	ssc04914: Progesterone-mediated oocyte maturation	12	−0.66	−1.61	0.012	0.508	0.701	56	Tags = 42%, list = 11%, signal = 46%
4	ssc00140: Steroid hormone biosynthesis	4	−0.85	−1.58	0.012	0.529	0.814	44	Tags = 75%, list = 9%, signal = 81%
5	ssc04621: NOD-like receptor signaling pathway	13	−0.62	−1.56	0.019	0.49	0.861	49	tags = 46%, list = 10%, signal = 50%
6	ssc05221: Acute myeloid leukemia	11	−0.63	−1.54	0.034	0.478	0.901	133	tags = 73%, list = 26%, signal = 96%
7	ssc05218: Melanoma	10	−0.64	−1.51	0.049	0.562	0.955	100	tags = 50%, list = 19%, signal = 61%
8	ssc03040: Spliceosome	9	−0.67	−1.5	0.039	0.524	0.961	101	tags = 44%, list = 20%, signal = 54%
9	ssc04640: Hematopoietic cell lineage	19	−0.53	−1.48	0.055	0.55	0.982	120	tags = 58%, list = 23%, signal = 73%
10	ssc04210: Apoptosis	10	−0.61	−1.48	0.066	0.504	0.984	158	tags = 70%, list = 31%, signal = 99%
11	Ssc05214: Glioma	11	−0.61	−1.47	0.052	0.467	0.985	100	tags = 45%, list = 19%, signal = 55%
12	ssc04012: ErbB signaling pathway	13	−0.56	−1.45	0.076	0.539	0.999	102	tags = 38%, list = 20%, signal = 47%
13	ssc05020: Prion diseases	8	−0.64	−1.44	0.07	0.533	1	65	tags = 50%, list = 13%, signal = 56%
14	ssc04666: Fc gamma R-mediated phagocytosis	12	−0.56	−1.4	0.098	0.641	1	135	tags = 67%, list = 26%, signal = 88%
15	ssc04650: Natural killer cell mediated cytotoxicity	19	−0.52	−1.4	0.087	0.605	1	89	tags = 42%, list = 17%, signal = 49%
16	ssc00650: Butanoate metabolism	5	−0.72	−1.39	0.088	0.581	1	6	tags = 20%, list = 1%, signal = 20%
17	ssc00410: Beta-Alanine metabolism	5	−0.71	−1.38	0.076	0.603	1	6	tags = 20%, list = 1%, signal = 20%
18	ssc04660: T cell receptor signaling pathway	18	−0.49	−1.35	0.112	0.689	1	122	tags = 56%, list = 24%, signal = 70%
19	ssc04370: VEGF signaling pathway	8	−0.61	−1.35	0.152	0.664	1	122	tags = 63%, list = 24%, signal = 81%
20	ssc05219: Bladder cancer	10	−0.56	−1.34	0.132	0.648	1	147	tags = 60%, list = 29%, signal = 82%
21	ssc04920: Adipocytokine signaling pathway	12	−0.53	−1.34	0.146	0.634	1	78	tags = 42%, list = 15%, signal = 48%
22	ssc04114: Oocyte meiosis	12	−0.54	−1.34	0.132	0.608	1	56	tags = 25%, list = 11%, signal = 27%
23	ssc00750: Vitamin B6 metabolism	2	−0.86	−1.33	0.09	0.6	1	72	tags = 100%, list = 14%, signal = 116%
24	ssc00260: Glycine, serine and threonine metabolism	4	−0.71	−1.32	0.131	0.597	1	128	tags = 75%, list = 25%, signal = 99%
25	ssc04960: Aldosterone-regulated sodium reabsorption	5	−0.69	−1.32	0.139	0.579	1	106	tags = 60%, list = 21%, signal = 75%
26	ssc00910: Nitrogen metabolism	3	−0.76	−1.29	0.164	0.646	1	91	tags = 67%, list = 18%, signal = 81%
27	ssc05220: Chronic myeloid leukemia	14	−0.5	−1.27	0.206	0.69	1	172	tags = 57%, list = 34%, signal = 84%
28	ssc04115: P53 signaling pathway	12	−0.51	−1.27	0.197	0.687	1	156	tags = 75%, list = 30%, signal = 105%
29	ssc04630: Jak-STAT signaling pathway	19	−0.44	−1.22	0.234	0.813	1	105	tags = 42%, list = 20%, signal = 51%
30	ssc00640: Propanoate metabolism	9	−0.53	−1.22	0.23	0.798	1	61	tags = 22%, list = 12%, signal = 25%
31	ssc05213: Endometrial cancer	10	−0.51	−1.21	0.248	0.787	1	133	tags = 50%, list = 26%, signal = 66%
32	ssc00591: Linoleic acid metabolism	4	−0.66	−1.19	0.27	0.829	1	44	tags = 75%, list = 9%, signal = 81%
33	ssc05215: Prostate cancer	17	−0.44	−1.19	0.243	0.811	1	115	tags = 35%, list = 22%, signal = 44%
34	ssc00280: Valine, leucine and isoleucine degradation	11	−0.49	−1.18	0.269	0.826	1	157	tags = 45%, list = 31%, signal = 64%
35	ssc00620: Pyruvate metabolism	7	−0.54	−1.17	0.285	0.818	1	239	tags = 100%, list = 47%, signal = 185%
36	ssc00010: Glycolysis/Gluconeogenesis	12	−0.47	−1.17	0.26	0.796	1	243	tags = 83%, list = 47%, signal = 155%
37	ssc04150: MTOR signaling pathway	7	−0.56	−1.17	0.291	0.78	1	42	tags = 29%, list = 8%, signal = 31%
38	ssc00250: Alanine, aspartate and glutamate metabolism	5	−0.6	−1.16	0.302	0.79	1	6	tags = 20%, list = 1%, signal = 20%
39	ssc00511: Other glycan degradation	4	−0.63	−1.16	0.312	0.772	1	195	tags = 100%, list = 38%, signal = 160%
40	ssc05212: Pancreatic cancer	13	−0.45	−1.16	0.304	0.754	1	172	tags = 54%, list = 34%, signal = 79%
41	ssc00604: Glycosphingolipid biosynthesis	4	−0.63	−1.15	0.299	0.755	1	195	tags = 100%, list = 38%, signal = 160%
42	ssc00052: Galactose metabolism	3	−0.66	−1.12	0.338	0.835	1	52	tags = 33%, list = 10%, signal = 37%
43	ssc00520: Amino sugar and nucleotide sugar metabolism	6	−0.54	−1.11	0.353	0.831	1	116	tags = 50%, list = 23%, signal = 64%
44	ssc04070: Phosphatidylinositol signaling system	6	−0.55	−1.11	0.358	0.813	1	100	tags = 67%, list = 19%, signal = 82%
45	ssc04662: B cell receptor signaling pathway	12	−0.45	−1.11	0.336	0.798	1	120	tags = 58%, list = 23%, signal = 74%
46	ssc00500: Starch and sucrose metabolism	4	−0.6	−1.1	0.37	0.809	1	57	tags = 50%, list = 11%, signal = 56%
47	ssc05014: Amyotrophic lateral sclerosis (ALS)	5	−0.57	−1.09	0.404	0.812	1	49	tags = 40%, list = 10%, signal = 44%
48	ssc00310: Lysine degradation	4	−0.59	−1.09	0.381	0.795	1	149	tags = 75%, list = 29%, signal = 105%
49	ssc04144: Endocytosis	18	−0.41	−1.08	0.384	0.814	1	26	tags = 17%, list = 5%, signal = 17%
50	ssc00533: Keratan sulfate biosynthesis	2	−0.69	−1.07	0.389	0.801	1	162	tags = 100%, list = 32%, signal = 146%
51	ssc04623: Cytosolic DNA-sensing pathway	8	−0.47	−1.07	0.4	0.791	1	30	tags = 25%, list = 6%, signal = 26%
52	ssc00340: Histidine metabolism	5	−0.55	−1.06	0.397	0.806	1	149	tags = 60%, list = 29%, signal = 84%
53	ssc00980: Metabolism of xenobiotics by cytochrome P450	9	−0.45	−1.04	0.442	0.835	1	44	tags = 33%, list = 9%, signal = 36%
54	ssc00270: Cysteine and methionine metabolism	5	−0.53	−1.04	0.478	0.824	1	245	tags = 100%, list = 48%, signal = 190%
55	ssc04330: Notch signaling pathway	6	−0.5	−1.03	0.473	0.833	1	4	tags = 17%, list = 1%, signal = 17%
56	ssc00330: Arginine and proline metabolism	10	−0.43	−1.03	0.447	0.824	1	174	tags = 50%, list = 34%, signal = 74%
57	ssc05223: Non-small cell lung cancer	7	−0.47	−1.01	0.487	0.844	1	89	tags = 43%, list = 17%, signal = 51%
58	ssc04720: Long-term potentiation	8	−0.46	−1.01	0.48	0.84	1	89	tags = 25%, list = 17%, signal = 30%
59	ssc04110: Cell cycle	19	−0.36	−1	0.472	0.832	1	220	tags = 58%, list = 43%, signal = 98%
60	ssc04730: Long-term depression	13	−0.39	−1	0.464	0.822	1	34	tags = 15%, list = 7%, signal = 16%
61	ssc05211: Renal cell carcinoma	13	−0.4	−1	0.467	0.812	1	193	tags = 62%, list = 38%, signal = 96%
62	ssc04912: GnRH signaling pathway	13	−0.38	−0.98	0.507	0.842	1	122	tags = 31%, list = 24%, signal = 39%
63	ssc00983: Drug metabolism	5	−0.5	−0.97	0.515	0.838	1	44	tags = 60%, list = 9%, signal = 65%
64	ssc00450: Selenoamino acid metabolism	3	−0.58	−0.97	0.547	0.84	1	176	tags = 67%, list = 34%, signal = 101%
65	ssc00071: Fatty acid metabolism	8	−0.43	−0.95	0.556	0.856	1	244	tags = 75%, list = 48%, signal = 141%
66	ssc00020: Citrate cycle (TCA cycle)	9	−0.41	−0.95	0.533	0.845	1	309	tags = 100%, list = 60%, signal = 247%
67	ssc03018: RNA degradation	2	−0.6	−0.93	0.583	0.866	1	206	tags = 100%, list = 40%, signal = 166%
68	ssc00603: Glycosphingolipid biosynthesis	6	−0.44	−0.93	0.58	0.866	1	195	tags = 83%, list = 38%, signal = 133%
69	ssc00053: Ascorbate and aldarate metabolism	3	−0.54	−0.92	0.599	0.86	1	239	tags = 100%, list = 47%, signal = 186%
70	ssc04020: Calcium signaling pathway	20	−0.33	−0.92	0.578	0.85	1	29	tags = 10%, list = 6%, signal = 10%
71	ssc00510: *N*-Glycan biosynthesis	8	−0.41	−0.91	0.607	0.863	1	306	tags = 100%, list = 60%, signal = 244%
72	ssc00903: Limonene and pinene degradation	3	−0.54	−0.91	0.631	0.851	1	239	tags = 100%, list = 47%, signal = 186%
73	ssc03320: PPAR signaling pathway	13	−0.36	−0.91	0.598	0.843	1	78	tags = 31%, list = 15%, signal = 35%
74	ssc04142: Lysosome	16	−0.34	−0.89	0.623	0.867	1	195	tags = 75%, list = 38%, signal = 117%
75	ssc05210: Colorectal cancer	13	−0.35	−0.89	0.599	0.857	1	42	tags = 15%, list = 8%, signal = 16%
76	ssc04622: RIG-I-like receptor signaling pathway	12	−0.35	−0.87	0.626	0.867	1	49	tags = 25%, list = 10%, signal = 27%
77	ssc04320: Dorso-ventral axis formation	2	−0.57	−0.87	0.674	0.86	1	34	tags = 50%, list = 7%, signal = 53%
78	ssc04130: SNARE interactions in vesicular transport	2	−0.57	−0.87	0.676	0.857	1	123	tags = 50%, list = 24%, signal = 66%
79	ssc00230: Purine metabolism	6	−0.42	−0.85	0.68	0.869	1	21	tags = 17%, list = 4%, signal = 17%
80	ssc00380: Tryptophan metabolism	4	−0.46	−0.85	0.699	0.865	1	239	tags = 75%, list = 47%, signal = 139%
81	ssc04910:Insulin signaling pathway	16	−0.32	−0.84	0.688	0.859	1	104	tags = 25%, list = 20%, signal = 30%
82	ssc00190: Oxidative phosphorylation	17	−0.31	−0.84	0.666	0.85	1	317	tags = 94%, list = 62%, signal = 238%
83	ssc00051: Fructose and mannose metabolism	4	−0.46	−0.84	0.697	0.848	1	69	tags = 25%, list = 13%, signal = 29%
84	ssc04614: Renin-angiotensin system	3	−0.5	−0.84	0.715	0.839	1	143	tags = 33%, list = 28%, signal = 46%
85	ssc00860: Porphyrin and chlorophyll metabolism	3	−0.49	−0.83	0.709	0.836	1	238	tags = 67%, list = 46%, signal = 124%
86	ssc00350: Tyrosine metabolism	5	−0.43	−0.83	0.692	0.833	1	263	tags = 80%, list = 51%, signal = 163%
87	ssc00561: Glycerolipid metabolism	6	−0.38	−0.79	0.733	0.87	1	239	tags = 83%, list = 47%, signal = 154%
88	ssc00030: Pentose phosphate pathway	5	−0.41	−0.79	0.744	0.868	1	57	tags = 20%, list = 11%, signal = 22%
89	ssc04540: Gap junction	11	−0.32	−0.77	0.764	0.884	1	134	tags = 27%, list = 26%, signal = 36%
90	ssc04916: Melanogenesis	14	−0.29	−0.75	0.787	0.892	1	145	tags = 29%, list = 28%, signal = 39%
91	ssc00562: Inositol phosphate metabolism	7	−0.35	−0.75	0.775	0.884	1	100	tags = 43%, list = 19%, signal = 53%
92	ssc03050 Proteasome:	4	−0.4	−0.74	0.814	0.887	1	97	tags = 25%, list = 19%, signal = 31%
93	ssc00600: Sphingolipid metabolism	5	−0.36	−0.71	0.821	0.919	1	107	tags = 40%, list = 21%, signal = 50%
94	ssc00630: Glyoxylate and dicarboxylate metabolism	2	−0.46	−0.7	0.907	0.916	1	142	tags = 50%, list = 28%, signal = 69%
95	ssc04512: ECM-receptor interaction	11	−0.29	−0.69	0.853	0.916	1	8	tags = 9%, list = 2%, signal = 9%
96	ssc00531: Glycosaminoglycan degradation	4	−0.35	−0.65	0.899	0.946	1	240	tags = 75%, list = 47%, signal = 140%
97	ssc05216: Thyroid cancer	8	−0.29	−0.63	0.904	0.948	1	133	tags = 38%, list = 26%, signal = 50%
98	ssc04520: Adherens junction	10	−0.26	−0.61	0.902	0.957	1	170	tags = 40%, list = 33%, signal = 59%
99	ssc00564: Glycerophospholipid metabolism	5	−0.3	−0.58	0.94	0.969	1	219	tags = 60%, list = 43%, signal = 104%
100	ssc00360: Phenylalanine metabolism	5	−0.26	−0.51	0.972	0.992	1	219	tags = 60%, list = 43%, signal = 104%
101	ssc04270: Vascular smooth muscle contraction	14	−0.18	−0.46	0.988	0.998	1	34	tags = 7%, list = 7%, signal = 7%
102	ssc04350: TGF-beta signaling pathway	14	−0.18	−0.45	0.982	0.99	1	172	tags = 36%, list = 34%, signal = 52%

**Table 7:** Gene sets enriched in phenotype control group (CG).

No.	Pathway	Size	ES	NES	NOM *p*-val	FDR *q*-val	FWER *p*-val	Rank at Max	Leading edge
1	ssc05320: Autoimmune thyroid disease	15	0.64	1.97	0.003	0.034	0.058	139	tags = 87%, list = 27%, signal = 115%
2	ssc04940: Type I diabetes mellitus	16	0.59	1.86	0.003	0.053	0.165	139	tags = 88%, list = 27%, signal = 116%
3	ssc05330: Allograft rejection	17	0.54	1.72	0.015	0.116	0.458	139	tags = 82%, list = 27%, signal = 109%
4	Ssc04530: Tight junction	17	0.52	1.67	0.013	0.141	0.628	2	tags = 12%, list = 0%, signal = 11%
5	ssc04260: Cardiac muscle contraction	13	0.55	1.65	0.03	0.132	0.683	107	tags = 46%, list = 21%, signal = 57%
6	ssc05412: Arrhythmogenic right ventricular cardiomyopathy	12	0.56	1.57	0.051	0.196	0.853	155	tags = 75%, list = 30%, signal = 105%
7	ssc02010: ABC transporters	2	0.83	1.34	0.136	0.6	0.998	89	tags = 100%, list = 17%, signal = 121%
8	ssc05340: Primary immunodeficiency	7	0.54	1.3	0.179	0.619	0.999	61	tags = 43%, list = 12%, signal = 48%
9	ssc05217: Basal cell carcinoma	5	0.56	1.25	0.241	0.668	1	114	tags = 60%, list = 22%, signal = 76%
10	ssc04740: Olfactory transduction	3	0.67	1.22	0.259	0.664	1	172	Tags = 100%, list = 34%, signal = 150%
11	ssc04120: Ubiquitin mediated proteolysis	8	0.47	1.18	0.271	0.683	1	32	Tags = 25%, list = 6%, signal = 26%
12	ssc03010: Ribosome	17	0.37	1.17	0.257	0.657	1	328	Tags = 100%, list = 64%, signal = 268%
13	ssc01040: Biosynthesis of unsaturated fatty acids	2	0.73	1.15	0.336	0.634	1	11	Tags = 50%, list = 2%, signal = 51%
14	ssc05012: Parkinson’s disease	17	0.35	1.13	0.302	0.633	1	337	Tags = 100%, list = 66%, signal = 282%
15	ssc05332: Graft-versus-host disease	15	0.36	1.1	0.367	0.646	1	139	Tags = 80%, list = 27%, signal = 107%
16	ssc00590: Arachidonic acid metabolism	11	0.39	1.08	0.352	0.634	1	55	Tags = 36%, list = 11%, signal = 40%
17	ssc04360: Axon guidance	12	0.36	1.05	0.393	0.653	1	56	Tags = 33%, list = 11%, signal = 37%
18	ssc04310: Wnt signaling pathway	14	0.35	1.03	0.462	0.66	1	132	Tags = 43%, list = 26%, signal = 56%
19	ssc04340: Hedgehog signaling pathway	3	0.55	1	0.479	0.671	1	114	Tags = 67%, list = 22%, signal = 85%
20	ssc00982: Drug metabolism	12	0.35	0.99	0.455	0.651	1	40	Tags = 25%, list = 8%, signal = 26%
21	ssc04080: Neuroactive ligand-receptor interaction	17	0.29	0.94	0.525	0.706	1	43	tags = 29%, list = 8%, signal = 31%
22	ssc00830: Retinol metabolism	7	0.39	0.94	0.524	0.682	1	16	Tags = 29%, list = 3%, signal = 29%
23	ssc00565: Ether lipid metabolism	4	0.43	0.84	0.686	0.811	1	295	Tags = 100%, list = 58%, signal = 233%
24	ssc05222: Small cell lung cancer	14	0.26	0.79	0.718	0.848	1	51	Tags = 21%, list = 10%, signal = 23%
25	ssc00480: Glutathione metabolism	6	0.34	0.78	0.746	0.832	1	339	Tags = 100%, list = 66%, signal = 291%
26	ssc05310: Asthma	11	0.27	0.77	0.741	0.806	1	139	Tags = 73%, list = 27%, signal = 98%
27	ssc00563: Glycosylphosphatidylinositol (GPI)-anchor biosynthesis	2	0.49	0.77	0.769	0.776	1	263	Tags = 100%, list = 51%, signal = 204%
28	ssc00601: Glycosphingolipid biosynthesis	4	0.33	0.68	0.846	0.857	1	164	Tags = 75%, list = 32%, signal = 109%

**Table 8:** Information on the primers used for qRT-PCR.

Confirmation objects	Gene symbol	Primer sequence (5′→3′)	Amplicon length (bp)	Ta (°C)	GenBank No.
*Reference gene*	*ACTB*	TCTGGCACCACACCTTCT	114	60	DQ178122
		TGATCTGGGTCATCTTCTCAC			

	*TBP*	GATGGACGTTCGGTTTAGG	124	60	DQ178129
		AGCAGCACAGTACGAGCAA			

	*TOP2B*	AACTGGATGATGCTAATGATGCT	137	60	AF222921
		TGGAAAAACTCCGTATCTGTCTC			

*Up gene*	*RETN*	AGTGCGCTGGCATAGACTGG	197	60	NM_213783
		CATCCTCTTCTCAAGGTTTATTTCC			

	*ADAM17*	TTGAGGAAGGGGAAGCC	158	56	NM_001099926
		ACGGAGCCCACGATGTT			

	*GPNMB*	GAGACCCAGCCTTCCTT	130	51.2	NM_001098584
		TTGCTTTCTATCGCTTTGTA			

	*CHRM1*	CGCTGGTCAAGGAGAAGAA	185	56	NM_214034
		GCACATGGGGTTGATGGT			

	*ALDH2*	AAACTGCTCTGCGGTGGA	181	56	NM_001044611
		CGTACTTGGAATTGTTGGCTC			

	*IL6*	GTCGAGGCTGTGCAGATTAG	101	56	NM_214399
		GCATTTGTGGTGGGGTTAG			

*Down gene*	*KLRK1*	TGATGTGATAAACCGTGGTG	107	56	NM_213813
		TGGATCGGGCAAGGAAA			

	*DUOX2*	CCCTTCTTCAACTCCCTG	158	51.2	NM_213999
		CAAAAGTTCTCATAGTGGTGC			

	*OAS2*	GACACGGCTGAAGGTTT	291	51.2	NM_001031796
		TGGCACGTCCCAAGACT			

	*KCNAB1*	AAGGGAGAAAACAGCAAAAC	176	56	NM_001105294
		AACCTGAATGGCACCGA			
